# Effects of Soil Salinity on the Expression of Bt Toxin (Cry1Ac) and the Control Efficiency of *Helicoverpa armigera* in Field-Grown Transgenic Bt Cotton

**DOI:** 10.1371/journal.pone.0170379

**Published:** 2017-01-18

**Authors:** Jun-Yu Luo, Shuai Zhang, Jun Peng, Xiang-Zhen Zhu, Li-Min Lv, Chun-Yi Wang, Chun-Hua Li, Zhi-Guo Zhou, Jin-Jie Cui

**Affiliations:** 1 Key Laboratory of Crop Physiological Ecology and Production Management, Ministry of Agriculture, and Department of Agronomy, Nanjing Agricultural University, Nanjing, Jiangsu, China; 2 State Key Laboratory of Cotton Biology, Institute of Cotton Research, Chinese Academy of Agricultural Sciences, Anyang, Henan, China; Institut Sophia Agrobiotech, FRANCE

## Abstract

An increasing area of transgenic *Bacillus thuringiensis* (Bt) cotton is being planted in saline-alkaline soil in China. The Bt protein level in transgenic cotton plants and its control efficiency can be affected by abiotic stress, including high temperature, water deficiency and other factors. However, how soil salinity affects the expression of Bt protein, thus influencing the control efficiency of Bt cotton against the cotton bollworm (CBW) *Helicoverpa armigera* (Hübner) in the field, is poorly understood. Our objective in the present study was to investigate the effects of soil salinity on the expression of Bt toxin (Cry1Ac) and the control efficiency of *Helicoverpa armigera* in field-grown transgenic Bt cotton using three natural saline levels (1.15 dS m^-1^ [low soil-salinity], 6.00 dS m^-1^ [medium soil-salinity] and 11.46 dS m^-1^ [high soil-salinity]). We found that the Bt protein content in the transgenic Bt cotton leaves and the insecticidal activity of Bt cotton against CBW decreased with the increasing soil salinity in laboratory experiments during the growing season. The Bt protein content of Bt cotton leaves in the laboratory were negatively correlated with the salinity level. The CBW populations were highest on the Bt cotton grown in medium-salinity soil instead of the high-salinity soil in field conditions. A possible mechanism may be that the relatively high-salinity soil changed the plant nutritional quality or other plant defensive traits. The results from this study may help to identify more appropriate practices to control CBW in Bt cotton fields with different soil salinity levels.

## Introduction

Soil salinization is an important impediment to sustainable agricultural development [1,2]. The total area of saline land in nearly 100 countries is approximately 1010 million ha, accounting for approximately 25% of the total land area. Because of improper irrigation or poor drainage, approximately 300,000 ha of cultivated land worldwide are affected by secondary salinization [3]. In China, saline-alkaline soil occupies an area of 37 million ha, accounting for 4.9% of the arable land. The recent increase in the area of salinized land, which has reached 9 million ha and represents 6.6% of the total cultivated land area, endangers crop production [4]. Cotton is one of the most important cash crops worldwide and a pioneer crop on salinized land; thus, the cultivation of cotton on saline or alkaline land is a preferred alternative strategy for future agricultural production on a limited land area to guarantee grain crop output [5]. Developing techniques for cotton cultivation on saline or alkaline land has become therefore an important focus of agricultural research in China.

The cotton bollworm (CBW), *Helicoverpa armigera* (Hübner), is one of the most destructive insect pests of cotton production in China. Since the late 1990s, transgenic *Bacillus thuringiensis* (Bt) cotton has been planted on a large scale in China to control CBW populations. In 2015, more than 3.7 million ha of transgenic Bt cotton, 96% of the total cotton-growing area, were cultivated in China [6]. Bt cotton has successfully suppressed CBW populations, leading to a drastic decrease in the use of insecticides on cotton and increased yields [7,8], and the widespread adoption of transgenic crops benefits natural predators and enhances associated ecosystem services, including the control of other arthropod pests [9–13]. However, the large-scale planting of transgenic cotton has also led to ecological problems, including pest status evolution, in which secondary pests in conventional systems become the primary pest in Bt cotton-growing systems [13–14]. The Yellow River Delta is one of the largest cotton-producing regions in China. This region contains 0.35 million ha of salt-affected land for cotton planting, and the portion of cultivated land affected by secondary salinization has increased annually [15,16]. Therefore, transgenic Bt cotton will be increasingly planted in this saline-alkali land.

The soil’s physical and chemical properties can affect plant growth and nutrition which, in turn, can affect plant attractiveness and susceptibility to insect herbivores [17–19]. Host plant nutrition can also modify the plant's reaction to insects and its susceptibility to insect feeding, creating conditions that are more or less favourable to insects, which can have varying effects on the herbivore density [18,20]. For example, increasing the salinity in a salt marsh decreased the plant foliar nitrogen but increased the planthopper density [21]. These processes are called bottom-up (host plant quality) forces and usually influence the fitness and population dynamics of herbivores [22–29].

Adverse environmental conditions, such as extreme temperatures [30–32], drought and waterlogging [33,34], substantially reduce the Bt protein content in transgenic Bt cotton and thus affect the control efficiency against CBW. Previous studies have investigated NaCl stress in indoor conditions or salt ponds, focusing on the seedling stage of cotton and have shown a negative correlation between the soil salinity and Bt protein content in cotton leaves [35,36]. Other studies have examined salinity stress-induced changes in the control efficiency of Bt cotton against CBW [35,37]. However, the stress in controlled conditions from NaCl has certain differences from the natural saline stress encountered in the field [38–41]; thus, the results obtained in the laboratory may be subject to certain restrictions under real conditions for application to cotton production in practice. To discuss the responses of transgenic Bt cotton to soil salinity, the relationship between the salinity level, Bt protein content and control efficiency in transgenic cotton need to be investigated under field conditions.

In this study, field experiments were conducted over the entire cotton growing season in natural saline soil, and the effects of salinity on the temporal and spatial variation of Bt protein content and the efficiency of transgenic Bt cotton in controlling the CBW were studied. These experiments provide a basis for production practices of transgenic Bt cotton against the CBW in saline soils.

## Materials and Methods

### Experimental design

In 2013 and 2014, field experiments were conducted at the experimental cotton station of Nanjing Agricultural University (120°46′E, 33°20′N) located in the Dafeng Basic Seed Farm in Dafeng, Jiangsu Province, China. The soil had three natural salinity levels (low, medium and high at 1.15, 6.00 and 11.46 dS m^−1^, respectively). Transgenic Bt cotton (GK19) and its near-isoline variety (Simian-3) were chosen in this study. The salt conductivities in the soils were measured with consistent texture and nutrient levels. Plots with baseline levels of low (1.15 dS m^−1^), medium (6.00 dS m^−1^) and high (11.46 dS m^−1^) soil salinities were selected for this experiment. The plots with low and medium salinity conductivities had been previously planted with rice for 5 and 2 consecutive years, respectively, and the plot with high salinity conductivity had not been planted with either rice or cotton. Seeds of Bt cotton and non-Bt cotton were provided by the Institute of Plant Protection, Soil and Fertilizer of the Hubei Academy of Agricultural Sciences, Wuhan, China, and sown in the selected plots with three different salinity levels at a density of 45,000 hm^-2^ on Apr 28, 2013 and May 4, 2014. For each salinity level, 6 plots including 3 replicates for every cotton variety were randomly assigned. Each plot was 20 m in length and 10 m in width. No chemical pesticides were applied to the experimental plots during the entire cotton-growing season.

### Insects

CBW larvae were provided by the insect laboratory of the Cotton Research Institute (CRI) of the Chinese Academy of Agricultural Sciences (CAAS), Anyang, Henan Province, China. Bollworms were collected from cotton grown on the CRI CAAS farm (36.13°N, 114.85°E) in September 2012 and reared in the laboratory under a set temperature (27 ± 0.5°C), relative humidity (75 ± 5%) and photoperiod (14 h:10 h, light: dark) with an artificial diet. One-day-old CBW larvae were used for the bioassays.

### Soil salinity content measurements

Soil samples were collected at a depth of 20 cm with a punch at the seedling, budding, flowering and bolling stages. Approximately 200 g of soil was collected from each plot at five different positions. The soil samples were mixed and air-dried at ambient temperature, ground and sifted, and the soil salt content was measured according to previously published methods [42].

Air-dried soil (100 g) was weighed in a 1000-ml conical flask with a wide mouth, 500 ml of CO_2_-free water was added, and the flask was plugged. The flask was then placed on a vibrating table for 20 min (120 rpm min^−1^) and subsequently centrifuged for 5 min at 3000 rpm min^-1^ until the supernatant became transparent. A 100-ml subsample of the supernatant was extracted, transferred into a 250-ml conical flask of known weight, distilled in a water bath, and then dried in the oven at 105°C for 4 h. The dried sample was allowed to cool in a dryer for approximately 30 min and was then weighed on an analytical balance. The flask was placed in an oven for 2 h of further drying at 105°C, cooled and weighed until a constant weight was achieved. The difference between the two measured weights could not exceed 0.0003 g. From these measurements, the soluble salt content was calculated:

$$\text{Content of soluble salt}=\frac{{\text{m}}_{1}-{\text{m}}_{0}}{\text{m}}\times 100$$

where the content of soluble salt is the mass fraction of the total soluble salt in the soil (%), m_1_ is the total mass of the beaker and salt (g), m_0_ is the mass of the empty beaker (g), m is the sample mass equivalent to the volume of liquid to be determined (g), and the multiplication by 100 converts the value into the content per hectogram.

### Quantification of the CBW population in an experimental field plot

Twenty plants were selected at each site in a continuous block of two rows, and 5 sites were sampled in each plot. Therefore, 100 plants were selected to investigate the CBW community in each plot. Each plant was visually examined, and the numbers of CBW larvae and eggs were counted separately. The open field survey started in early June and lasted until late September, with a sampling interval of 5 days.

### Feeding bioassays using Bt cotton leaves derived from soils with different salinities

The youngest fully expanded main stem leaves (the fourth leaf from the shoot apex) were collected at the seedling stage (emergence period of the second-generation bollworms), and the sympodial leaves from the upper fruiting branches were collected at the budding stage (emergence period of the third-generation bollworms) and the flowering and bolling stages (emergence period of the fourth-generation bollworms). Ten leaves were sampled from each plot at each sampling time, and each leaf was placed in a plastic bioassay box. Five 1-day-old CBW larvae were placed in the bioassay box and kept at (27 ± 0.5°C) with a relative humidity of (75 ± 5%) and a 14 h:10 h (light: dark) photoperiod for 5 d, and the number of live larvae was then recorded. The corrected mortality rate of the larvae on transgenic Bt cotton, which was obtained based on the mortality rate on non-Bt cotton as the control, was calculated using the formula [43]

$$\text{Corrected mortality of larvae }\left(\text{\%}\right)\;=\frac{{\text{x}}_{\text{t}}-{\text{x}}_{0}}{1-{\text{x}}_{0}}\times 100$$

where x_t_ is the mortality of the larvae on Bt transgenic cotton and x_0_ is the mortality of the larvae on non-transgenic cotton. The control efficiency of the transgenic cotton is represented by the corrected mortality of the CBW larvae, where a higher corrected mortality indicates a higher control efficiency.

### Bt protein content detection

Samples were collected for Bt protein detection in the same manner as those for the feeding bioassays. Five leaves were collected from each plot, immediately frozen, and stored at -80°C for analysis.

An enzyme-linked immunosorbent assay was used to detect the Bt protein content. Fresh cotton tissue (0.4 g) was ground in liquid nitrogen, 4 ml of 0.01 mol l^−1^ PBS (phosphate-buffered saline) was added, and the sample was placed on a table concentrator (200 rpm min^-1^) overnight. The supernatant was collected after centrifugation at 8000 × g, and the Bt protein content was detected using the QuantiPlate^™^ Kit for *Cry1Ab*/*Cry1Ac* (EnviroLogix, Portland, ME, USA) according to the manufacturer’s instructions.

### Data analyses

Analysis of variance was performed to examine the effects of soil salinity on dynamics of the CBW population, control efficiency and Bt protein content. The means were separated using Duncan’s multiple range tests at the 5% probability level. The correlations between the soil salinity, control efficiency against CBW and Bt protein content were performed. All the data were analyzed using SPSS 17.0 software (SPSS, Chicago, IL, USA).

## Results

### Dynamics of soil salinity

**Fig 1:**
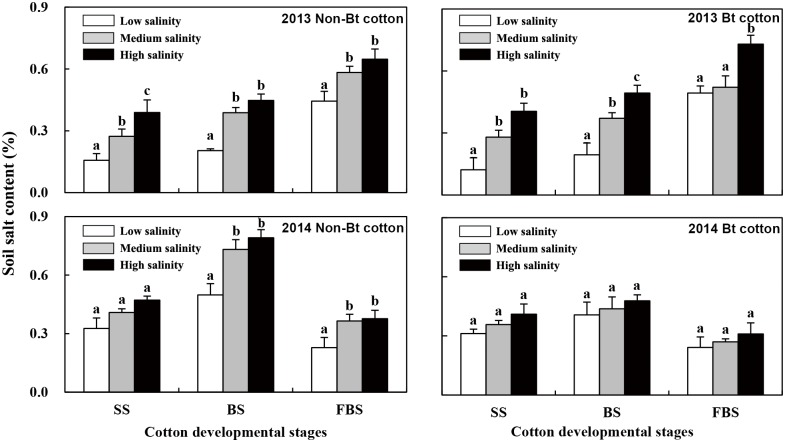
Measurement of the soil salt content in fields of Bt cotton and non-Bt cotton in 2013 and 2014. SS, seedling stage; BS, budding stage; FBS, flowering and bolling stage. Lowercase letters above the bars indicate significant differences among the salinity treatments (*p* < 0.05).

Fig 1 shows the dynamics of the soil salinity throughout the different cotton growth stages. The soil salt content gradually increased from the seedling stage to the flowering and bolling stages in 2013 and presented a trend of first increasing and then decreasing in 2014. In the non-Bt cotton fields, the soil salinity levels of the low-, medium- and high-salinity soils were significantly different in both years, except for in the seedling stage in 2014. In the Bt cotton fields, the differences in soil salinity during the same growth stages were noticeably different among the low-, medium- and high-salinity soils in 2013, but these differences were not significant in 2014.

### Dynamics of the CBW population

#### CBW eggs

**Fig 2:**
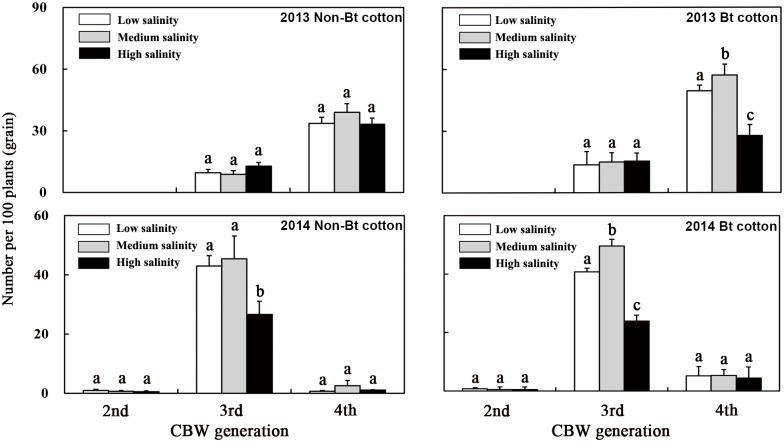
Effect of soil salinity on the number of CBW eggs on Bt cotton plants in 2013 and 2014. 2nd, period of second-generation CBW occurrence; 3rd, period of third-generation CBW occurrence; 4th, period of fourth-generation CBW occurrence. Lowercase letters above the bars indicate significant differences among the salinity treatments (*p* < 0.05).

There were three peaks in the numbers of accumulated CBW eggs on Bt cotton and non-Bt cotton (Fig 2). The highest number of eggs was observed during the CBW emergence periods of the fourth generation in 2013 and the third generation in 2014. With the increasing soil salinity, the number of CBW eggs on the cotton plants increased when the salinity of the soil increased from low to medium but declined at the high level.

During the period of CBW occurrence, the number of CBW eggs per 100 plants was investigated. The largest number of eggs was found on the Bt cotton plants grown in medium-salinity soil, and the lowest number was found on plants grown in high—salinity soil. The number of eggs on the plants grown in medium-salinity soil was 15.5% (F = 7.773, *p* = 0.049) higher in 2013 and 21.5% (F = 33.034, *p* = 0.005) higher in 2014 than those on the plants grown in low-salinity soil, and the number of eggs on the cotton plants grown in high-salinity soil was 44.1% (F = 43.552, *p* = 0.003) lower in 2013 and 41.4% (F = 145.637, *p* < 0.001) lower in 2014 than those on the plants grown in low-salinity soil.

In 2014, there were no significant differences between the transgenic Bt cotton and non-Bt cotton in terms of the number of CBW eggs per 100 plants, either in the plants grown in soils with the same salinity level or at the same growth stage.

#### CBW larvae

**Fig 3:**
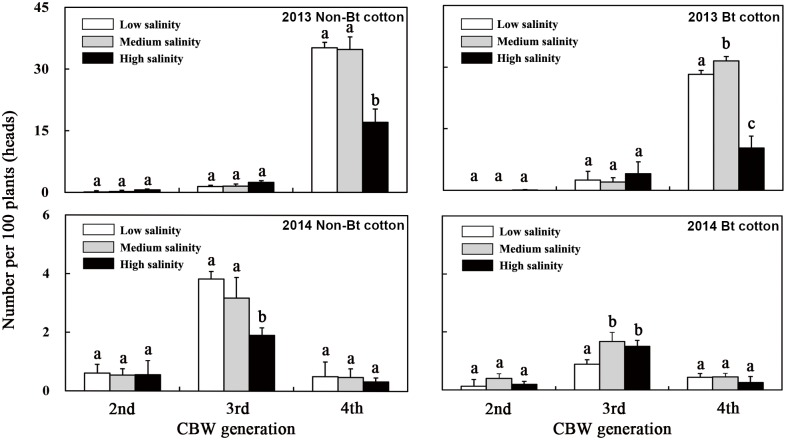
Effect of soil salinity on the number of CBW larvae on Bt cotton plants in 2013 and 2014. 2nd, period of second-generation CBW occurrence; 3rd, period of third-generation CBW occurrence; 4th, period of fourth-generation CBW occurrence. Lowercase letters above the bars indicate significant differences among the salinity treatments (*p* < 0.05).

The CBW larval populations on the Bt cotton and non-Bt cotton peaked during the fourth generation in 2013 and the third generation in 2014. The number of CBW larvae in 2013 was significantly higher than that in 2014 (Fig 3).

During the peak emergence period of the fourth generation of the CBW populations in 2013 in the transgenic Bt cotton field, the number of larvae per 100 plants grown in the medium-salinity soil was 11.8% (F = 15.235, *p* = 0.017) higher than that in the low-salinity soil; in the high-salinity soil, the number of larvae per 100 plants was 63.5% (F = 171.554, *p* < 0.001) lower than that in the low-salinity soil. Over the peak emergence period of the third-generation CBW in 2014, the number of CBW larvae per 100 plants grown in the medium- and high-salinity soils was 87.6% (F = 19.465, *p* = 0.012) and 68.5% (F = 29.287, *p* = 0.006) higher, respectively, than the number in the low-salinity soil (Fig 3).

On the non-Bt cotton, although the number of CBW larvae per 100 plants was not obviously different between the low- and medium-salinity soils, these numbers were both higher than the number of larvae per 100 plants grown in high-salinity soil. The number of CBW larvae per 100 Bt cotton plants in the low-salinity soils was 19.8% (F = 46.832, *p* = 0.002) lower in 2013 and 76.8% (F = 261.368, *p* < 0.001) lower in 2014 when compared with the non-Bt cotton plants, although there were relatively small differences between them in the medium- and high-salinity soils.

### Efficiency of transgenic Bt cotton in controlling CBW larvae

**Fig 4:**
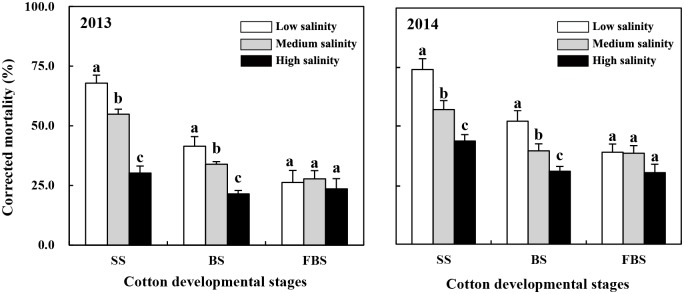
Corrected mortality of larvae on the leaves of Bt cotton plants at three developmental stages. SS, seedling stage; BS, budding stage; FBS, flowering and bolling stage. Lowercase letters above the bars indicate significant differences among the salinity treatments *(p* < 0.05).

The control efficiency of the transgenic Bt cotton against CBW decreased significantly with increasing soil salinity at the seedling and budding stages (Fig 4). At the seedling stage, the control efficiency of the transgenic Bt cotton against CBW larvae in the medium- and high-salinity soils were 19.3% (F = 27.645, *p* = 0.006) and 55.6% (F = 161.700, *p* < 0.001) lower, respectively, and 23.0% (F = 28.564, *p* = 0.006) and 41.0% (F = 111.049, *p* < 0.001) lower, respectively, in 2014, when compared with the low-salinity soil. At the budding stage, the control efficiencies in the medium- and high-salinity soils were 18.3% (F = 9.118, *p* = 0.039) and 48.2% (F = 46.980, *p* = 0.002) lower, respectively, compared to the low-salinity soil in 2013 and 24.1% (F = 17.872, *p* = 0.013) and 40.7% (F = 55.954, *p* = 0.002) lower, respectively, in 2014.

### Bt protein content in transgenic Bt cotton

**Fig 5:**
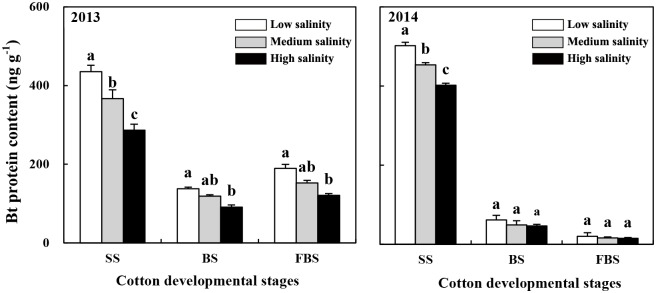
Bt protein content in Bt cotton leaves at three developmental stages. SS, seedling stage; BS, budding stage; FBS, flowering and bolling stage. Lowercase letters above the bars indicate significant differences among the salinity treatments (*p* < 0.05).

The Bt protein content in the leaves of the transgenic Bt cotton significantly decreased with increasing soil salinity (Fig 5). At the seedling stage, the Bt protein content in plants grown in medium- and high-salinity soils were 15.8% (F = 18.438, *p* = 0.013) and 34.2% (F = 137.780, *p* < 0.001) lower than in the plants grown in low-salinity soil in 2013, respectively, and 9.6% (F = 62.693, *p* = 0.001) and 19.9% (F = 284.573, *p* < 0.001) lower, respectively, in 2014. In 2013, the Bt protein content in the plants grown in high-salinity soils was lower than in the plants grown in the low-salinity soil at the budding stage and the flowering and bolling stages; however, in 2014, the Bt protein contents were not different between the plants grown in soils with different salinities during these stages.

### Correlation between soil salinity, Bt protein content and control efficiency in transgenic Bt cotton

The relationship between the soil salinity, Bt protein content and control efficiency was analysed. A significant correlation was observed between the soil salinity and the Bt protein content in transgenic Bt cotton and between the Bt protein content and control efficiency against CBW (S1 and S2 Tables). Soil salinity was significantly negatively correlated with Bt protein content in 2013, but no significant correlation was observed in 2014.

## Discussion

The soil physical and chemical properties and abiotic factors (mechanical wounds, ultraviolet radiation, environmental stress, etc.) [44–46] can affect plant growth and nutrition [18–20]. Host plant nutrition can also modify the plant’s reaction to insects, which can have varied effects on herbivore densities [18,20]. Some studies have indicated that high temperatures [30–32], drought and waterlogging [37,22,34], nitrogen nutrition [47,48] and salinity stress [38–41] can affect the control efficiency and Bt protein content of transgenic Bt cotton. In this study, we focused on the influence of soil salinity on the Bt protein content and control efficiency of Bt cotton and the change in the pest population in the field during the growing season.

The expression of the Bt protein in transgenic cotton is regulated by the growth and development of the plant [49–51]. The laboratory data shows that the Bt protein production by transgenic cotton (sampled from the field in three periods) was highest in the plants grown in low-salinity soil, followed by those of the medium and then high salinities. The Bt protein content in the cotton leaves was negatively correlated with soil salinity, which is consistent with the findings of previous research conducted in the laboratory using different NaCl concentrations [35–36]. Jiang et al. [35] reported that the Bt protein content in the leaves of 99B and SGK9708-41 were 44% and 62% higher under non-stress conditions than under 200 mmol L^−1^ NaCl stress, respectively. Iqbal et al. [36] reported an inverse relationship between the Bt protein content in the leaves and buds and the soil NaCl concentration at 90, 120 and 150 days after seedling emergence for cotton grown in a soil: manure: sand (2:1:1) mixture irrigated with a NaCl solution. In addition, the control efficiency of transgenic cotton against CBW larvae decreased when cotton was stressed by growth in saline soils, which is consistent with the results of Li, who observed a discernible decline in insect resistance of transgenic Bt cotton(29312) grown under NaCl stress in the greenhouse [52]. However, the results are not in line with those of Jiang et al. [35] and Luo et al. [37], who found that the control efficiency was not affected at the seedling stage in the laboratory. The difference could be due to some factors, e.g., the protein content in the leaves is high in the early seedling stage and the degree of decrease is not enough to cause a decline in insect resistance, the use of leaves versus whole plants, differences in varieties used, densities of CBW in bioassays, combined effects of salinity and waterlogging [37], or differences in the experimental setup (indoor versus field).

In the present study, the population dynamics of CBW were investigated on field-grown cotton under soil salinity stress. The response pattern obtained indoors was consistent with the field survey results of CBW populations in low and medium soil salinities, but not those in high-salinity conditions. Salt stress adversely affects the biomass production of cotton plants and decreases the leaf area, stem thickness and shoot and root weights, which ultimately results in decreased cotton yield [42]. Excessive salt in the soil leads to a series of physiological and biochemical metabolic disorders in cotton plants, mainly because of osmotic effects (dehydration), nutritional imbalance and the toxicity of salt ions (Na^+^ and Cl_-_) [53–55]. At present, growing evidence suggests that both top-down and bottom-up forces affect the natural herbivore populations, and those interactions may occur among the limiting factors [56]. Vegetation can affect herbivores directly by influencing their performance and survival and indirectly by mediating the effects of predators on herbivore populations [57,58]. Plant choice is often first made by the mother during oviposition, although immature individuals of some species may make additional choices between staying at the same location, moving on the same plant, or moving to a different plant [20]. Both Dethier [59] and Chew [60] believed that a strong selection pressure causes females to be selective in their choices, which can also affect herbivore diversity and abundance [61]. Despite that Bt protein production was the lowest under high soil-salinity conditions in our case, the cotton growth potential may be greatly reduced compared to that in low and medium-salinity soils [62]. For example, the leaf area, number of fruiting branches and boll retention abscission rate of cotton plants grown in high-salinity soil were noticeably reduced, and the cotton nutrition probably changed [63, 64]. These changes may cause the inconsistency between the laboratory and field survey results of the CBW eggs and larvae populations in the high-salinity field.

Previous research has involved experiments under controlled or semi-controlled conditions, whereas our research was conducted under natural salinity conditions in the field, which is conducive to understanding the patterns of variation in the Bt protein content in field with different salinities. With increasing soil salinity from weak to moderate, the CBW population in the transgenic Bt cotton increased, and the mortality and Bt protein content declined. Even if the Bt protein content was negatively correlated with the salinity level, it could not be concluded that the CBW population would increase in the relatively high soil salinity field. In other words, the Bt protein content may play a secondary role in mediating the bottom-up effects on the CBW population, and the population size may be related to salinity-mediated changes in the plant nutritional quality or other plant defensive traits. This mechanism needs to be further studied in future work.

## Supporting Information

S1 Table Correlation between the soil salinity and Bt protein content in the leaves of transgenic Bt cotton (Pearson correlation). n = 9; * Significant at the *p* = 0.05 level; ** Significant at the *p* = 0.01 level.
(DOCX)

S2 Table Correlation between the control efficiency against CBW and Bt protein content in transgenic Bt cotton under different soil salinities (Pearson correlation). n = 9; * Significant at the *p* = 0.05 level; ** Significant at the *p* = 0.01 level.
(DOCX)
