# Supplementary material for: Effects of Soil Salinity on the Expression of Bt Toxin (Cry1Ac) and the Control Efficiency of Helicoverpa armigera in Field-Grown Transgenic Bt Cotton
Source: PLoS One. 2017 Jan 18;12(1):e0170379. doi: 10.1371/journal.pone.0170379 (PMC5242435; doi:10.1371/journal.pone.0170379)
Supplement: S1 Table — n = 9; * Significant at the p = 0.05 level; ** Significant at the p = 0.01 level. (DOCX) [file pone.0170379.s001.docx]

**S1 Table. Correlation between soil salinity and Bt protein content in the leaves of transgenic Bt cotton (Pearson correlation).**

| **Years** | **Seedling stage** | **Budding stage** | **Flowering and bolling stage** |
| --- | --- | --- | --- |
| 2013 | - 0.945** | - 0.888** | - 0.846** |
| 2014 | - 0.588 | - 0.600 | - 0.198 |

n = 9; * Significant at the *p* = 0.05 level; ** Significant at the *p* = 0.01 level.
