# Supplementary material for: Effects of Soil Salinity on the Expression of Bt Toxin (Cry1Ac) and the Control Efficiency of Helicoverpa armigera in Field-Grown Transgenic Bt Cotton
Source: PLoS One. 2017 Jan 18;12(1):e0170379. doi: 10.1371/journal.pone.0170379 (PMC5242435; doi:10.1371/journal.pone.0170379)
Supplement: S2 Table — n = 9; * Significant at the p = 0.05 level; ** Significant at the p = 0.01 level. (DOCX) [file pone.0170379.s002.docx]

**S2 Table. Correlation between control efficiency against cotton bollworm and Bt protein content in transgenic Bt cotton under different soil salinities (Pearson correlation).**

| **Years** | **Seedling** | **Budding** | **Flowering and bolling** |
| --- | --- | --- | --- |
| 2013 | 0.989** | 0.927** | 0.749* |
| 2014 | 0.986** | 0.806** | 0.593 |

n = 9; * Significant at the *p* = 0.05 level; ** Significant at the *p* = 0.01 level.
